# Adhesive Plaster for the Cure of Certain External Diseases and Lesions

**Published:** 1908-03

**Authors:** M. B. Hutchins

**Affiliations:** Atlanta, Ga. Chief of Skin and Cancer Service. Presbyterian Hospital, Baptist Tabernacle Infirmary and Wesley Memorial Hospital, etc.


### ADHESIVE PLASTER FOR THE CURE OF CERTAIN EXTERNAL DISEASES AND LESIONS.

BY M. B. HUTCHINS, M. D., ATLANTA, GA.

Chief of Skin and Cancer Service, Presbyterian Hospital, Baptist Tabernacle Infirmary and Wesley Memorial Hospital, etc.

In the *New York Medical Journal* (LXXV, p. 322, February 22, 1902) I published a paper detailing the method and success in the treatment of boil and abscess cavities by the carbonized poultice to rapid, final recovery. For the patient's convenience in going about during the day, adhesive plasters were employed as a direct, closed dressing.

Since this publication the use of rubber adhesive has been extended with such striking success in the shortening of time of treatment and the minimum of scar left that it would seem a service to present the details to the profession.

Following the caustic or electrolytic destruction of a lesion, epitheliomatous or otherwise, the adhesive plaster has been closely fitted to the eschar and crust as they hardened sufficiently to obviate too free oozing. Constant contact of the adhesive plaster, renewed as soon as soiled, hastens the softening and separation of the eschar and shortens by many days the time required for healing. This is simply a nice and convenient substitute for an ordinary poultice.

Observing the healing began and progressed well under the plaster, as the edges of the eschar were loosened, a few cases have been dressed, the plaster directly on the wound and periphery in the same way, to the final healing of the lesion. The small amount of moisture stimulates granulation, which "levels up" the wound, epidermizing proceeding rapidly. The scar is limited to a very small size relative to the original lesion. I have found, however, that this method leaves a rather red or livid scar, and often telangiectases which evidences of rapid formation of granulation tissue disappear very slowly, often the large vessels persisting. My older method seems preferable. After the last of the eschar has separated and the wound is ready to granulate from the bottom, the patient has for daytime wear, a powder such as bismuth subnitrate, which forms a crust. Over this, as a poultice, the adhesive plaster is applied at night. The next morning the crust is found liquified, the wound is then dried, and the powder put on. Under this plan we control (with half or two-thirds of the time a dry hard dressing and the rest a moist condition from partial retention of attracted serum) the activity of the new vascular tissue, prevent injury from the prolonged presence of crust, and obtain a whiter scar.

After full healing, the scale crusts that may form from the bountiful epithelial supply can easily be loosened by the use of the plaster without injury to the new tissue. Often the effect upon these or other dry, hard conditions would be nil, save for the fact that the needed moisture comes from the retained skin secretions.

I might here briefly note another use of the plaster, although the subject will be elaborated in a later paper, and that is, as a substitute for the, often unreliable, salicylic acid plaster. Desiring the full effect of the drug, it is directly applied to the lesion and covered with plaster. The results are better than with the strongest commercial salicylic acid adhesive.

I have had little experience or success with the adhesive plaster for closure or narrowing of incised wounds, slipping under tension defeating the object.

Indolent ulcers will usually begin to granulate if covered with adhesive directly, under a process of serotactic action and stimulation, even to final scar where, as on the legs, the appearance does not count.

The most striking results of the use of the adhesive plaster method have been in the treatment of boils and subcutaneous abscesses. In the case of boils, the patient usually appears at the time when the entrance of infection is a break in the skin of perhaps one eighth inch or less, and the surrounding tissue in the maximum stage of tension and pain; necrosis has just begun to show in the formation of a core, and liquefaction is not initiated. The majority of these lesions occur on the back of men's necks, often too high to dress easily. Such of the "core" as is removable is picked out, no incision or none more than the diameter of the core is made. A solution of 95 percent. carbolic acid is swabbed in and the whole area is then covered with the adhesive plaster. This dressing prevents crusting and permits enough outflow from beneath its edges, apparently increases the serous flow and phagocytosis, suggesting somewhat the Bier theory. The dressings are changed when soiled. A poultice made with a 2 or 3 percent. carbolic acid solution may be used at night if preferred, the principle being much the same. At each change of dressing, loose parts of the core are removed and carbolic acid again swabbed in, never followed by alcohol. The phenol destroys many virulent cocci and hastens necrosis of dying tissue. As the dead tissue decreases by liquefaction and discharge, the serous flow increases, and granulations of giant size rapidly fill the cavity. While the discharge is very free, a small pad of cotton is first applied, then the adhesive plaster to cover the whole area. This to prevent too much leakage from beneath the adhesive plaster and its annoyance to the patient. When the big, soft granulations get up to the skin level the constant presence of the plaster may continue their growth to the formation of a hypertrophic scar. A dry dressing may best be employed, at least part of the time, at this stage.

One of the greatest advantages of the plaster method lies in its closing the infection sufficiently to prevent the usual "crop" of boils. In practically 100 percent. of cases, I can assure the patient that his boils will end with those he brings on his first visit. A last advantage is that having the slightest incision, or opening, the scar is often difficult to find.

Perhaps more brilliant have been the results in subcutaneous abscesses, the skin showing no breach, movable over a fluctuating mass. These abscesses have varied from the size of a bean to that of a small tomato. Without any surgical preparation a one eighth to one quarter inch puncture is quickly made and every particle of the pus is pressed out until blood and then serum appear from the distended capillaries. When pressure has quite well "milked the cavity dry," a small piece of cotton is put on and all covered with the adhesive plaster over a sufficient area to insure some sloppiness beneath. Serous flow is constant, the incision can not heal nor crust, infection rapidly yields, and granulation begins to repair the damage. Often the one emptying ends the infection, at most the larger abscesses may show a little pus in the serum at the change of the dressing on the next day, but usually collected serum is pressed out instead. Three or four days see the end of the process in the smaller cases, rarely over a week is needed in the larger. As the cavity fills with granulations the little incision tends to heal under the adhesive and must be probed open each day until fluid no longer appears, then a last little piece of adhesive plaster and dismissal, with practically no scar nor depression of the area.

The dressings seem to owe some of their effect to exclusion of air, as shown by the fact that I have never seen pustules develop in the skin around the opening, though covered by the plaster and more or less bathed in the discharge. But the serum and its opsonins do the best of the work, while spongy granulations rebuild the tissues.

A few cases follow, showing the duration and results of treatment:

Case I.—Mrs. J., aged twenty-nine. There was an epithelioma on the right side of the middle of the nose, about one-quarter of an inch in diameter. Lesion and borders were destroyed with deliquesced caustic potash. Bismuth subnitrate was applied to cover dark, soft eschar. The next day the area was depressed, hard and dry. A piece of zinc oxide adhesive plaster was applied to the area and periphery. On the following day the edges showed slight separation of eschar crust. At the end of a week it was quite loose. On the eleventh day the wound was nearly free from eschar, the edges were healing, and it was entirely clean on the thirteenth day. On the fifteenth day, all but a "pin point" was healed. When seen five days later for dismissal, the scar was not over two third size of cauterized area, but a bit too red. The adhesive plaster was constantly on save for intervals of renewal.

A patient treated with caustic potash and the eschar left exposed has often been from three to four weeks waiting for sufficient separation that we might learn if the treatment had been thorough enough to permit healing and demonstrate proper destruction of the disease.

Case II.—A split pea sized ordinary wart on a young lady's wrist was destroyed with caustic potash. No adhesive plaster was employed. Eleven days later the small eschar could not be removed without pain and bleeding. The dead tissue was soon out and the lesion healed with three or four days' use of adhesive plaster.

Case III.—Diffuse epithelioma of the left nasoface area. X rays could not be employed on account of the patient, Miss.H., aged sixty-two living at a distance. Area was about one by one and one-quatrer inches, superficial, with some cicatrix. Deliquesced caustic potash was employed. Adhesive plaster was applied closely the next day, and patient continuing this treatment at home. At the end of a week some edge points below canthus were healed, and the main lesion was nearly clean. On the sixteenth day, an irregular band of hypertrophic granulations was seen across lower part. To control this, the adhesive plaster was now used at night and the bismuth subnitrate application in the daytime—"half and half" treatment. Ten days later, the entire area healed, but a slight recurrence in edge. This was treated. Total time was about twenty-five days, patient managing the dressings nearly all this time.

Case IV.—Mr. C., fifty-two years of age. Caustic potash destruction of epithelioma on the right cheek. Patient had to return home before final healing. He came back within six weeks with the report that it healed, but "a sort of blister immediately followed" in centre. In the middle of the small, level scar, was a clean cut, appleseed sized, smooth, raw defect. A minute piece of cotton was put in and the adhesive plaster over it. On the next day the cotton was omitted and the zinc adhesive plaster applied directly. The patient was well by the fifth day after his return.

Case V.—Following prolonged treatment of various forms of an ulcer in the middle of the front of left leg, probably the result of a nonspecific, fat necrosis, the healing reached about one and one-quarter inch in width, level with scar and skin, moist, indolent, flabby, while any new scar readily broke down. This absolute condition of standstill and relapse continued over a month. Now, in the hope of stimulating free granulations and preventing any crust effect, the adhesive plaster was applied over the raw surface and edges. The next day the lesion was very sloppy. On the second day there was a little healing, less wet. There was a little seropus the first few days. On the sixth day firm healing of upper and lower ends was noticed. On the eighth day upper half was nearly well, the rest healing. These formed a firm, pearly white scar. On the eleventh day, a lentil sized raw point still remained. The patient was well in about fifteen days from beginning use of adhesive plaster, with a slight scar ridge.

Case VI.—Mrs. X., fifty-three years of age, had an old x ray burn beneath right eye, finally healed to point size of end of a toothpick under gauze covered with adhesive plaster, the dressing was changed daily. Last point indolent and moist and did not heal under adhesive plaster farther than to the size of a pin point. The adhesive plaster retained moisture, but the tissues were too indolent to fill the small hole with granulations. Healing ultimately took place after stimulation with silver nitrate.

Case VII.—Mr. T. had a furunculosis and abscesses of the arms recurrent attacks, for two or three years. The lesions occurred chiefly on the extensors of the arms from elbows to deltoid insertion. The primary development was in the subcutaneous tissue, a livid to red swelling, finger tip to two inches in area, would form. Puncture always disclosed pus. Even in the case of the larger lesions puncture never exceeded one quarter inch. In slightly over two months I treated six or eight of these by pressing free, after puncture, of pus until blood and serum followed. The smaller lesions required less than a week, the larger, up to two inches in area and fully one inch cavities, required from two to three weeks, but because the patient would sometimes remain away,with same dressing on from two to four days. Scars were practically imperceptible. This patient was in a bad general condition. He was unable to explain the recurrences and multiplication of the lesions, but often a very small point was neglected until it became large and contained as much as an ounce of pus. After the second day it was exceptional to find any pus, but the time of healing was prolonged because of the indolence of the tissues around the large cavities left by the destructive process. He had been treated by the usual free incision and dry dressing. Results of this were imperfect, progress slow and scars marked.

Case VIII.—Miss F. Beginning over two weeks previously to the present attack a small "blind boil" formed on the left cheek over facial artery. Patient used various home remedies. Upon examination, the lesion was a stiff infiltration somewhat wider than a half dollar and one-half inch thick. Central part was soft and fluctuating. One-eighth inch puncture was made through the skin. More than one-half ounce of pus was pressed out, then a little blood and serum. Dressing consisted of cotton with adhesive plaster. Upon removal of dressing on the next day, there was some free serous outflow, faintest evidence of pus. On the third the cotton was slightly stained, but about I drachm retained serum was let out. On the fourth day there was a little serum, infiltration was almost gone. It was necessary to probe open. Patient was dismissed on the fifth day with a healing puncture not larger than a match end.

Case IX.—Mrs. M. showed a small epithelioma in the middle of top of nose, destroyed with x rays. Bismuth dressing formed too much crust and pressed down, even when the adhesive plaster was kept on half the time. The plaster was applied directly over a bit of cotton as sole dressing. Healing and granulation began at once, the patient being dismissed in five days.

Case X.—Illustrating the treatment of a boil of carbuncular type: Lesion developed six days before on back of neck. Patient was in delicate health. When first examined there was a one-half walnut sized elevation, tense, red, painful; over necrotic mass were several small openings. He had been poulticing. There was a necrosing, perforated skin area one-half dime size, from which some pus could be pressed out. Treatment consisted in a one-quarter inch incision, enlarging central hole, part of core was still adherent. This was cleansed with a fifty per cent. volume of hydrogen peroxide solution, then covered with a pledget of cotton, finally with zinc adhesive plaster. The lesion was cleansed and redressed twice a day, pus and particles of core being removed as presenting. On the third day there was only a finger end sized cavity. A solution of 95 per cent. of phenol was used to swab out, and this was repeated daily until the cavity was clean. On the fourth day a necrotic skin area one-half dollar size remained, cavity extending to nuchal muscles. On the fifth day large granulations appeared, pus formation was very free. The last of the necrotic tissue was removed on the seventh day. On the eighth day the swelling was gone, and the wound granulated nicely. On the ninth day the wound was entirely granulated to top save one inch deep sinus and one smaller one. 1 to 1,000 bichloride gauge was loosely put in and covered with adhesive plaster, which form of dressing was used for several days. On the tenth day there was just a trace of pus, and on the eleventh day no pus. Oval skin wound was now one-eighth by two-third inch. This was dressed once a day with a little gauze and the covering adhesive plaster, so as to draw the edges toward each other. On the fifteenth day large vascular granulations with freely exuding serum filled the wound to skin level. Everything was well, save healing over the granular tissue. This required some days longer because of its having been allowed to grow too high and the patient's neglecting the condition.

This case was really one of a carbuncle. Final cleansing had required about ten days, filling of cavity to level with new tissue fifteen days. There was no new lesion, no infection around, simply steady progress to recovery. The patient attended to his business most of the time.

Case XI.—Typifying this treatment for a common furuncle of the neck is the following case: Mr. A. had a half walnut sized and extremely painful boil on the right side of the back of neck just below the occipital hair margin. There was a minute central necrosis. The boil was punctured, carbolic acid being used first and then adhesive plaster as a dressing, twice a day. There was rapid progress to recovery, no new boils formed, and the final scar showed the size of a match end.

The usual "surgical" incision of boils and carbuncles is not only painful, but affords a larger and wounded surface for absorption of infection. Packing with gauze is painful, the gauze acts as a foreign body, irritating, and checks granulation. This dressing is dry and plugs up the wound. Finally the healing takes much longer and the scar is often a disfigurement.

1015 Century Building.

